# Supplementary material for: Carbon subsurface traffic jam as driver for methane oxidation activity and selectivity on palladium surfaces
Source: Nat Commun. 2025 Aug 20;16:7755. doi: 10.1038/s41467-025-63088-9 (PMC12368142; doi:10.1038/s41467-025-63088-9)
Supplement: Supplementary file 1 — Supplementary Information [file 41467_2025_63088_MOESM1_ESM.pdf]

Supplementary Information - Carbon Subsurface Traffic Jam as Driver for Methane Oxidation Activity and Selectivity on Palladium Surfaces

Ulrike Küst[1,2,*], Rosemary Jones[1,3], Julia Prumbs[1], Alessandro Namar[4], Mattia Scardamaglia[3], Andrey Shavorskiy[3], Jan Knudsen[1,2,3,*]

[1]Division of Synchrotron Radiation Research, Lund University, Box 118, SE-221 00 Lund, Sweden

[2]NanoLund, Lund University, Box 118, SE-221 00 Lund, Sweden

[3]MAX IV Laboratory, Lund University, Box 118, SE-221 00 Lund, Sweden

[4]Physics Department, University of Trieste, via A. Valerio 2, Trieste 34127, Italy

*Corresponding authors: ulrike.kust@sljus.lu.se, jan.knudsen@sljus.lu.se

S1 Supplementary Methods

For these measurements a cylindrical sample was mounted on a transferrable 304L stainless steel sample plate. An PID controlled IR laser was used to heat the crystal and the temperature was monitored with a type K thermocouple spot welded to the side of the crystals to ensure a precise temperature measurement. The reading was done once every third second. Before the experiments the crystal was cleaned by $1\,\mathrm{kV}$ Ar^+ sputtering at $1 \times 10^{-5}\,\mathrm{mbar}$ pressure and $10\,\mathrm{mA}$ emission current for $20\,\mathrm{min}$ followed by annealing to $650\,^\circ\mathrm{C}$. The cleanliness of the surface was confirmed by XPS survey scans. The footprint of the beam on the sample is $60\,\mu\mathrm{m} \times 25\,\mu\mathrm{m}$ and the measured gas phase signal originates from a volume consisting of this footprint size and a height of $90\,\mu\mathrm{m}$. The HIPP-3 electron analyzer with a $0.8\,\mathrm{mm}$ slit was operated in fixed acquisition mode using a $8\,\mathrm{Hz}$ acquisition frequency. A pass energy of $100\,\mathrm{eV}$ was used for the surface spectra, while $200\,\mathrm{eV}$ was used during gas phase measurements such that the entire binding energy range (approximately 10% of the pass energy) could be covered by the electron analyzer in fixed acquisition mode.

Oxygen (5.0 N) and CH_4 (3.5 N) were used for the experiments. Commercial Pall gas cleaners (GLP2OXPVMM4 for O_2 and GLPSIPVMM4 for CH_4) were used on both gas lines. The gases were dosed with mass flow controllers (Brooks GF125). The stated flow values in sccm units refers to standard conditions of $20\,°\mathrm{C}$ and $14.696\,\mathrm{psia}$ $(1\,\mathrm{bar})$. The pressures stated in the paper were measured at the cell outlet with a Baratron capacitance gauge. The gas composition in the cell is followed by a Quadropole Mass Spectrometer which probes the gas composition in the first differential pumping stage of the electron analyzer, i.e. at a roughly $600\,\mathrm{\mu m}$ distance from the catalyst surface.

To prepare the sample, a Pd(100) single crystal $(6\,\mathrm{mm}$ diameter) was put through several oxidation and reduction cycles that roughened the surface, and, while changing color a few times (light grey, blue, green, black), the surface eventually turned dull. Investigations with Low Energy Electron Diffraction (LEED) showed a complete loss of crystallinity at the surface while Grazing Incidence Surface X-ray Diffraction showed both powder rings and Bragg peaks suggesting that a bulk crystalline phase prevails. Survey spectra were collected throughout the measurement to check for possible contaminations. Impurities were not found at any point.

Even though the bulk of our sample is still crystalline, we expect similar results on a Pd foil as the ones presented in this paper due to the structural similarities of the sub-surface region.

The probing depths are estimated from the photoelectron kinetic energy and the universal curve for the electron mean free path in matter to be $0.6\,\mathrm{nm}$ for the highest surface sensitivity and $1.5\,\mathrm{nm}$ for the more bulk sensitive measurement. This corresponds to the probing of approximately 3 and 8 atomic layers in a Pd(100) crystal, respectively.

Assuming that the sensitivity of the mass spectrometer (MS) is similar for all gas components, the measured intensities are scaled to the total pressure. Additionally, a constant background has been subtracted from the H_2 signal (c.f. Fig. S5) since we do not expect hydrogen formation in the presence of molecular oxygen and instead assume H_2O decomposition in the spectrometer. To estimate the partial pressures of the gas phase components measured in APXPS, a scaling factor was first applied to the curve-fitted intensities to account for the different photoionization cross sections of the O *1s* and C *1s* peaks. By dividing by the number of corresponding atoms in the probed molecule and scaling to the total pressure, partial pressures of all components were obtained.

All data analysis was performed in Igor Pro 8 using purpose-written scripts. Every spectrum was corrected for the analyzer transmission function and the binding energy axes were calibrated using the Fermi edge. Since the electron analyzer is more accurate at lower electron kinetic energies, the spectra measured at higher photon energies were aligned with those measured at lower photon energies by using the gas phase binding energies. Time alignment was done using the gas phase work function shift. Polynomial background subtraction was carried out for each spectrum by fitting a polynomial to the datapoints where no components were visible. To determine the datapoint range for fitting the background subtraction the sum spectrum of all time resolved data was used (to ensure that the background subtraction did not remove any weak components). After background subtraction and normalization for varying electron transmission through the gas phase, all spectra were Fourier transformed and then the first 50 harmonics were inverse Fourier transformed. The IFT image was then curve fitted with symmetric Voigt functions with as little free parameters as possible. For example, a common Lorentzian width was used for all components $(0.1\,\mathrm{eV}$ [1]).

Component widths and surface BEs were determined at the example of one spectrum in the

entire image and then kept constant for the curve fit of the time evolution. The binding energy that was used for fitting surface carbon was $283.9\,\mathrm{eV}$ while SSR carbon was positioned at $284.3\,\mathrm{eV}$. Surface oxygen was fitted with a binding energy of $529.15\,\mathrm{eV}$. The Pd $3p_{\frac{3}{2}}$ BE was allowed to vary as well as those of the gas phase components which can be found in Fig. S5.

In short, the Fourier analysis methodology (discussed in detail in refs [2, 3]) uses Fourier transformation to selectively analyze the part of the XPS signal that oscillates with the same frequency as the temperature modulation ($17\,\mathrm{mHz}$, c.f. Fig. 1 (m)) or multiples thereof as well as the static part. By discarding all other frequency components and by inverse Fourier transforming one can greatly improve the signal-to-noise ratio as Fig. 1 (e-h) demonstrates. For this dataset, Fourier analysis even provides better results than event averaging (c.f. ref. [4] for more details on the method) especially for the noisy O *1s* data (c.f. Panel (a)) as no lock-in signal needs to be found in the image.

S2 Supplementary Discussion

Inspection of Fig. 4 (e) shows that each methane turnover generates two H_2 or two H_2O molecules. As we measure in a flow reactor and continuously remove formed products, the total methane turnovers (black solid curve in fig. 4 (d)) are proportional to the sum of 2 x (p(H_2)+p(H_2O)). Methane turnovers to CO (reaction III in fig. 4 (e)) are proportional to p(CO) and methane decomposition turnovers are proportional to p(H_2)/2 (reaction V). The remaining H_2O not produced via reaction III can be calculated as (p(H_2O)-2p(CO)). The remaining H_2O comes from either reaction II in oxygen-rich conditions or a combination of reaction II and reaction IV in methane-rich conditions. Simultaneously, the CO_2 production can be balanced either by pathway I and II for O_2-rich conditions or solely by reaction pathway II for CH_4-rich conditions. The balancing factors for each reaction pathway that are needed to make everything match up directly give the turnovers for reaction I, II, and IV.

In a more mathematical explanation, the reaction pathways II, III, and V are based on the observations of CO_2, CO, and H_2, respectively. The existence of the pathways I and IV can be deduced according to the following discussion.

The number of moles N for each species j can be written as

$$N_j = N_{j,0} + \sum \nu_{ij}\chi_i \tag{S1}$$

where $N_{j,0}$ is the initial number of moles of species j, ν_{ij} the stochiometric coefficient of species j in reaction i, and χ_i the extent of reaction i. Since our species are measured in a flow reactor, $N_{j,0} = 0$ and we can write

$$N_j = \sum \nu_{ij}\chi_i \tag{S2}$$

which can then be used to formulate equations for each species. Thus, we obtain

$$N_{CO2} = 1 \cdot \chi_{II} + 1 \cdot \chi_I \tag{S3}$$

$$N_{CO} = 1 \cdot \chi_{III} \tag{S4}$$

$$N_{H2} = 2 \cdot \chi_V \tag{S5}$$

$$N_{H2O} = 2 \cdot \chi_{II} + 2 \cdot \chi_{III} + 2 \cdot \chi_{IV} \tag{S6}$$

$$N_C = 1 \cdot \chi_V + \chi_{IV} \tag{S7}$$

where the roman numerals refer to the reaction equations in Fig. 4 (e). Hence, we directly get

$$\chi_{III} = N_{CO} \qquad \text{and} \tag{S8}$$

$$\chi_V = 0.5 \cdot N_{H2}. \tag{S9}$$

We can assume that the pathways (I) and (IV) cannot coexist since their sum would be pathway (II) again. Thus, if pathway (IV) is present, we set $\chi_I = 0$ which leads to

$$\chi_{II} = N_{CO2} \qquad \text{and} \tag{S10}$$

$$\chi_{IV} = 0.5 \cdot N_{H2O} - N_{CO} - N_{CO2}. \tag{S11}$$

Likewise, if pathway (I) is present, then $\chi_{IV} = 0$ and we obtain

$$\chi_{II} = 0.5 \cdot N_{H2O} - N_{CO} \qquad \text{and} \tag{S12}$$

$$\chi_I = N_{CO2} - \chi_{II} \tag{S13}$$

$$= N_{CO2} - 0.5 \cdot N_{H2O} - N_{CO} \tag{S14}$$

which defines all desired reaction extents.

Possible sources of error during the measurement include such that are made due to the principle of measurement and such that result from the data analysis. An example of the first category is that the resolution of the beamline decreases for increasing photon energies. Even if this effect is small, it might have lead to the overlooking of small peaks that are only present deeper in the bulk.

Most of the errors made result from the data analysis, however. The most significant one is probably the direct comparison of MS and XPS data. Not only are the resulting signals measured at different locations in the chamber (XPS very localized at the sample surface, MS averaged over the whole chamber in the analyzer cone), they might also have different sensitivities to different gases that we did not calibrate for. Due to the fact that the MS averages over the whole chamber and is located a bit away from the sample, the gas signals measured in the spectrometer cannot experience sharp changes but are rather smoothed out. This can, for example lead to the over- or underestimation of carbon deposition effects using the hydrogen signal.

An error that results solely from XPS is due to the depth profiling technique. Since the measured photoelectrons from deeper layers decay exponentially within the material, a direct comparison of surface and bulk component intensities most likely leads to a significant underestimate of the amount of probed bulk atoms.

In the beginning of the analysis of the XPS raw data, an error is introduced to the intensity of the components due to the chosen normalization for changing gas attenuation and beam intensity

fluctuations.

During the FT analysis of the XPS raw data, an additional error is introduced to the curve shape (see detailed discussion in ref [3]) and probably the component intensity. The magnitude of this error is, however, difficult to estimate.

The subsequent curve fit to the IFT image leads to error bars in the intensity evolution of the individual components resulting from the accuracy of the curve fit.

As an extended discussion of the surface spectra, and to estimate the thickness of the carbon diffusion layer, we note that the probed catalyst volume in Fig. S3 (e,k) is fully emptied of carbon at lower temperatures. When increasing the probing depth to about $4\,\mathrm{nm}$, this is no longer the case (see Fig. S3 (f,l)). A constant non-zero carbon signal (at $283.9\,\mathrm{eV}$) is measured indicating that, while the topmost ($3\,\mathrm{nm}$) catalyst surface layers are periodically filled with and emptied from carbon, the deeper catalyst layers (deeper than $4\,\mathrm{nm}$) never reach zero carbon concentration.

An additional effect of the increasing probing depth is that the gas phase peaks seem to vanish. The reason is the use of higher photon energies. This leads to a higher electron kinetic energy which leads to a higher electron mean free path. Thus, the detected electrons can originate from deeper in the bulk. At the same time, the amount of gas and surface components probed remains the same. Thus, surface and gas component intensities are reduced in comparison. The reason for the strong CH_4 compared to the bulk carbon signal in Fig. 1 (l) is the methane concentration in front of the surface and the lower bulk carbon concentration.

Hydroxyls on palladium surfaces during methane oxidation are observed at temperatures similar to those in this experiment (e.g. at $450\,^{\circ}\mathrm{C}$ [5]) and are expected to appear in the O *1s* spectral region at around $531.6\,\mathrm{eV}$ or just between the oxide and the Pd $3p_{\frac{3}{2}}$ peak. Indeed, one can notice a small shoulder of the PdO peak in Fig. S1 (j) at higher binding energies that could indicate the presence of hydroxyls on the surface, and, thus, an onset of catalyst poisoning by water [6]. Unfortunately, due to the high time resolution and the resulting low signal-to-noise ratio we want to avoid drawing a conclusion from such a small difference.

A thorough discussion of the Pd *3d* spectral region cannot be done as both surface and bulk contributions of carbides, oxides and metallic Pd are expected within a $2\,\mathrm{eV}$ window. Hence, the curve fit becomes rather arbitrary. We show, however, Pd *3d* spectra and possible curve fits based on peak positions in the literature [7–11] at a few selected times in Fig. S8. The conclusions drawn from the discussion of the O *1s* and C *1s* spectra can be supported by the Pd *3d* spectra. This is, among others, the change between an oxide and a carbide covered surface. This trend can be seen by fitting the spectra with all peak intensities free to vary, however, when using the knowledge from O *1s* and C *1s* spectra, the first two fits (at $t = 15\,\mathrm{s}$ and $t = 25\,\mathrm{s}$) at $0.6\,\mathrm{nm}$ probing depth were restricted to a zero carbide intensity while the last two ($t = 40\,\mathrm{s}$ and $t = 50\,\mathrm{s}$) were restricted to a zero oxide intensity. An additional conclusion that can be drawn from the Pd *3d* spectra is that a thick oxide is never formed as a metallic Pd bulk signal is detected at all times. Also, a thick coke layer can be excluded as remaining Pd atoms are measured at all times.

S3 Supplementary Figures

Evolution of the O *1s* region followed with different probed depths of the catalyst during the repeated carbon deposition and oxidation cycles induced by temperature modulations

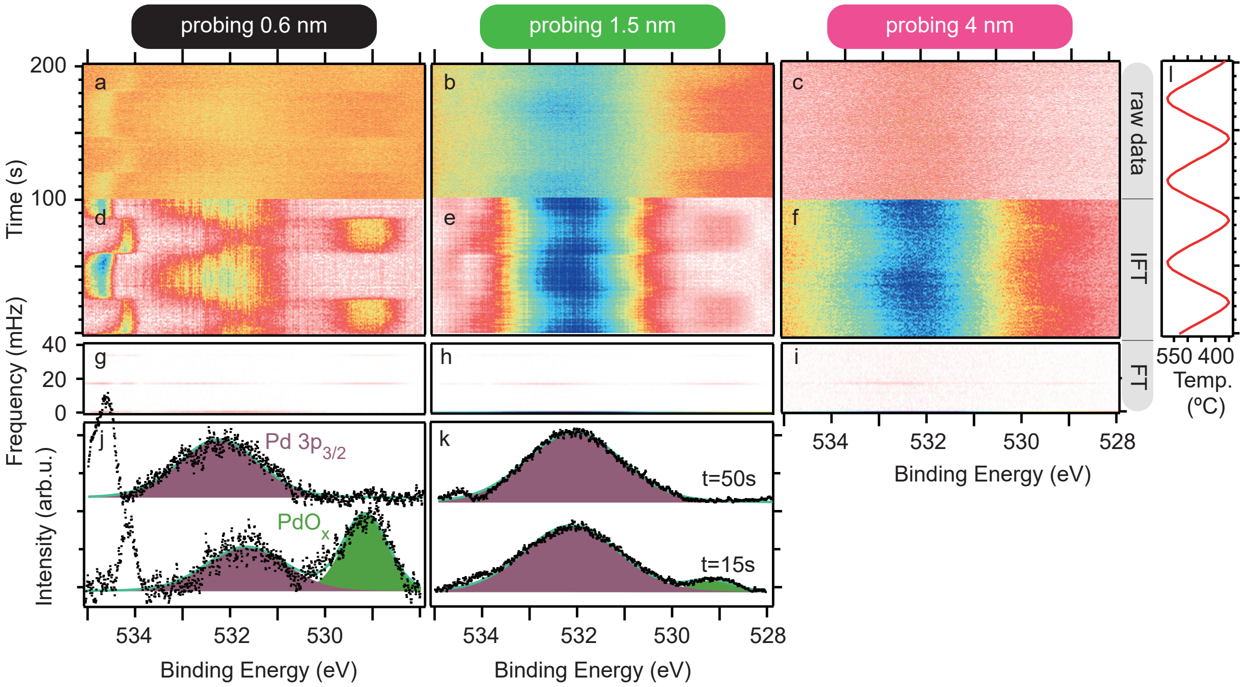

Figure S1: Measured O *1s* raw data (a,b,c), inverse Fourier transform of 50 harmonics (d,e,f) including the 0 Hz component of the Fourier transform as shown in (g,h,i), and examples of the curve fit to the IFT at two times (j,k) are shown together with the temperature modulation (l).

Evolution of the Pd $3d_{\frac{5}{2}}$ peak followed with different probed depths of the catalyst during the repeated carbon deposition and oxidation cycles induced by temperature modulations

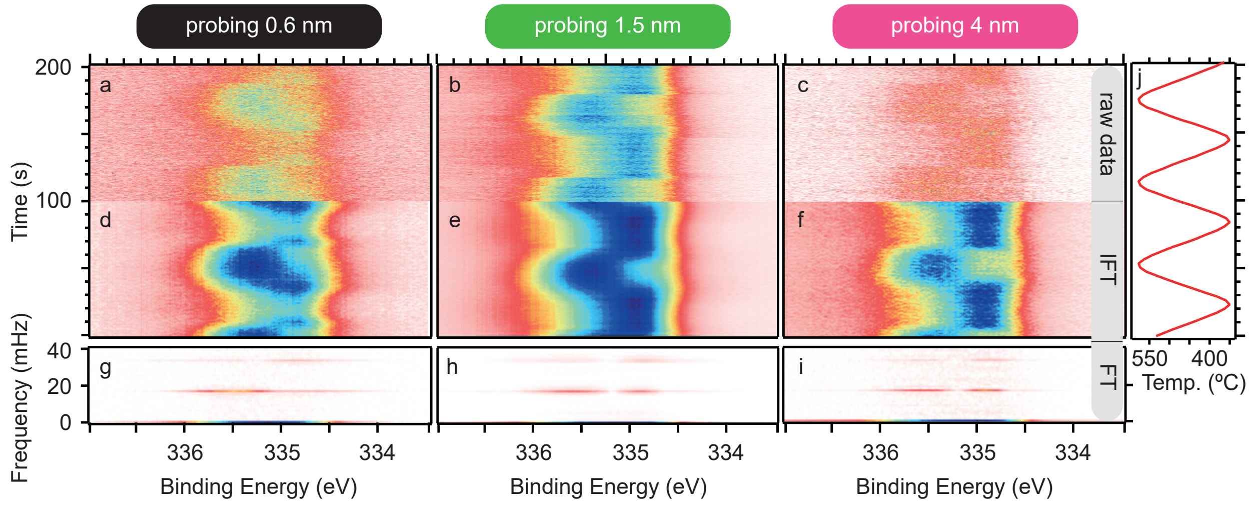

Figure S2: Measured Pd *3d* raw data (a,b,c), inverse Fourier transform of 50 harmonics (d,e,f) including the 0 Hz component of the Fourier transform as shown in (g,h,i), and the temperature modulation (j) are shown.

Carbon species buildup followed with different probed depths of the catalyst shown in data for the C *1s* core level

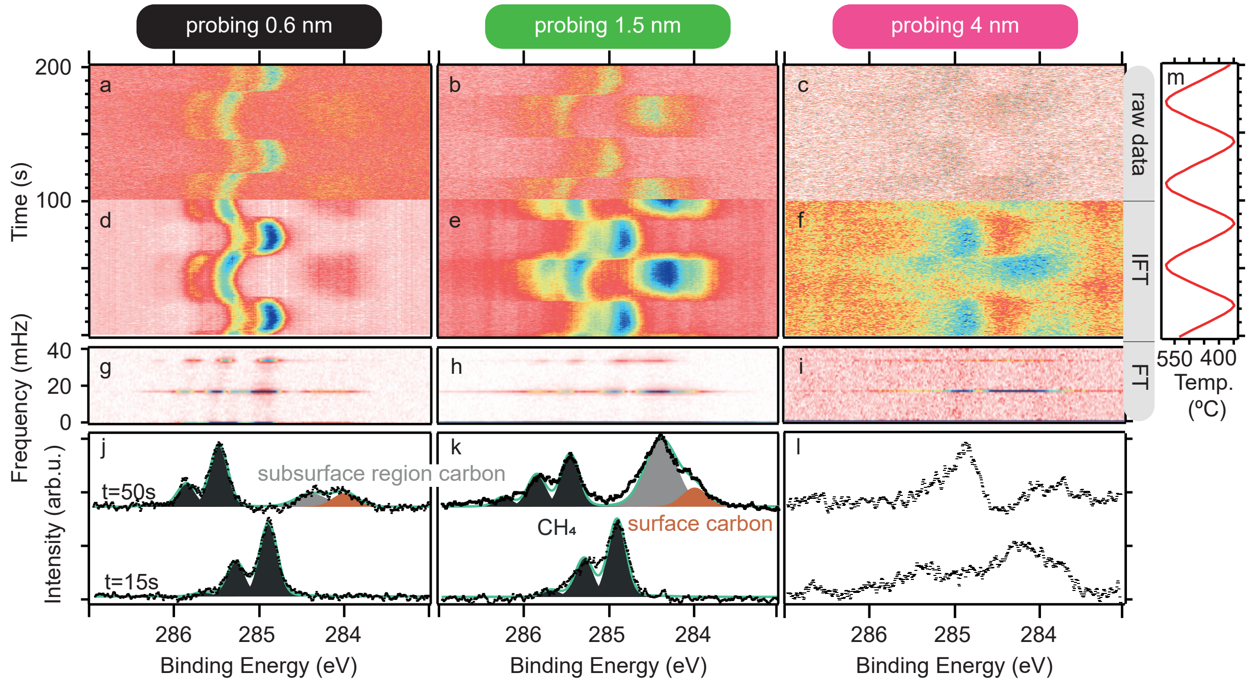

Figure S3: Measured C *1s* raw data (a,b,c), inverse Fourier transform of 50 harmonics (d,e,f) including the 0 Hz component of the Fourier transform as shown in (g,h,i), and examples of the curve fit to the IFT at two times (j,k) are shown together with the temperature modulation (m). Panel (l) shows extracts of Panel (f) at two times.

Gaseous product and reactant evolution during the temperature modulation followed in the C *1s* and O *1s* spectra

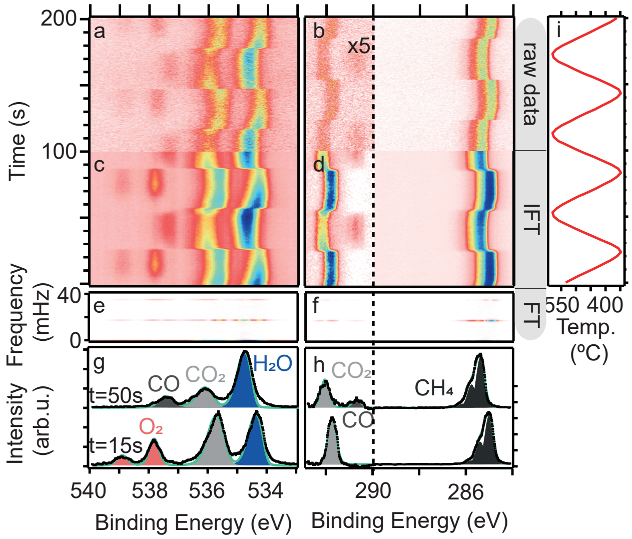

Figure S4: Measured raw data for the O *1s* (a) and C *1s* (b) gas phase spectra. The respective inverse Fourier transforms (IFT) of 50 harmonics are shown in (c) and (d) based on the Fourier transforms in (e,f). Panel (i) shows the temperature modulation signal applied to the catalyst. Examples of the curve fitting are shown at $t = 15\,\mathrm{s}$ and $t = 50\,\mathrm{s}$. The high BE side of the C *1s* spectra has been magnified 5 times for better visibility.

Evolution of gaseous product and reactant intensities and binding energies obtained from curve fitting of the XPS spectra and compared with mass spectrometry data

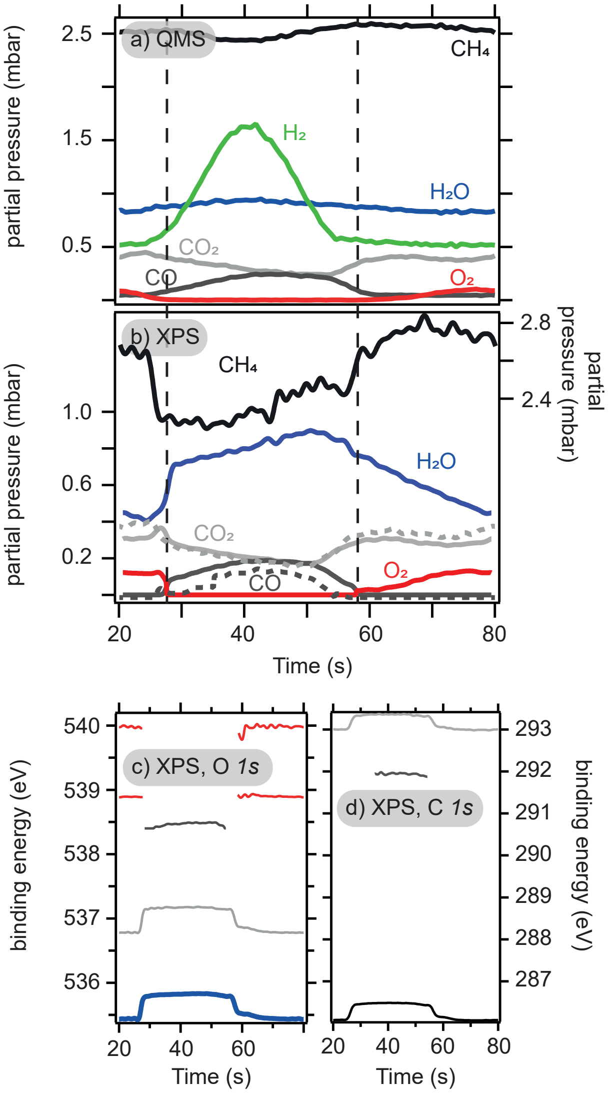

Figure S5: (a) Partial pressures of mass spectrometer data together with all those calculated from the curve fit to the APXPS gas phase spectra (b). The vertical dashed lines indicate the beginning and end of the O-MTL. The corresponding apparent binding energies of the O *1s* and C *1s* components are shown in (c,d).

Example of carbon not being fully removed from deeper catalyst layers when applying slightly different temperature modulations

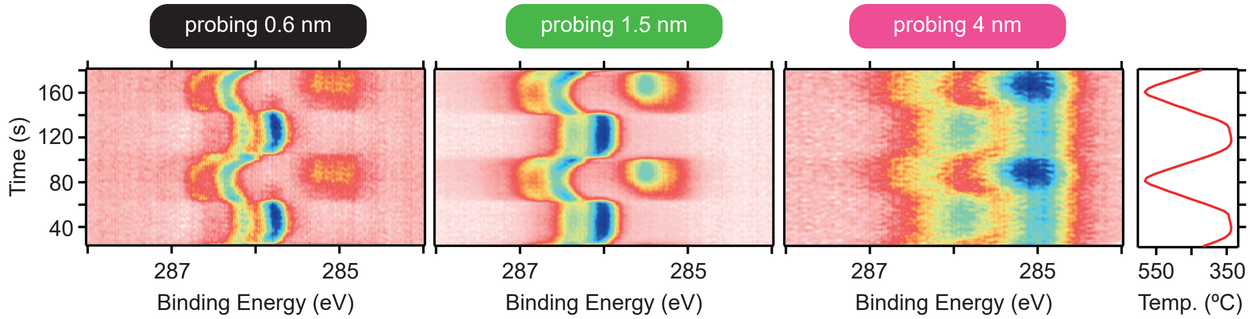

Figure S6: C *1s* spectra measured under similar conditions, just with a slightly larger temperature amplitude, i.e. $340\,°C$ $(50\,s)$ to $584\,°C$ $(30\,s)$. Carbon is fully removed from the surface but never from the bulk.

Evolution of Pd $3p_{\frac{3}{2}}$ and Pd $3d_{\frac{5}{2}}$ during the carbon deposition and oxidation cycle

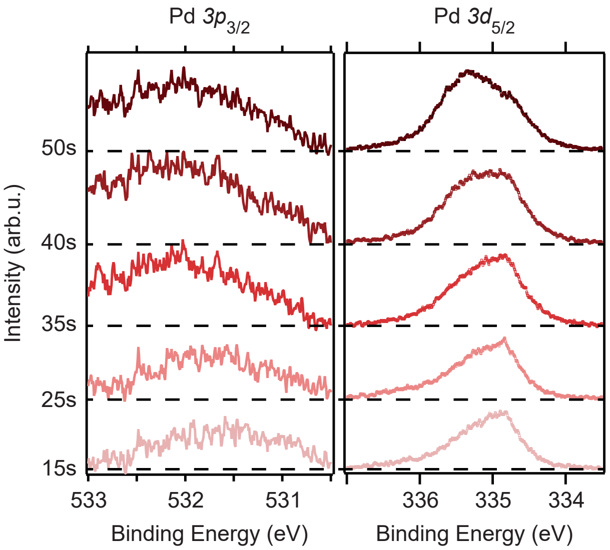

Figure S7: Measured Pd $3d_{\frac{5}{2}}$ and Pd $3p_{\frac{3}{2}}$ peaks measured at the highest surface sensitivity are shown at selected times. The peak shape development over time is rather similar, e.g. the main peak position shifts to higher binding energies and the entire peak becomes broader over time.

Evolution of the Pd $3d_{\frac{5}{2}}$ peak at two probing depths during the carbon deposition and oxidation cycle

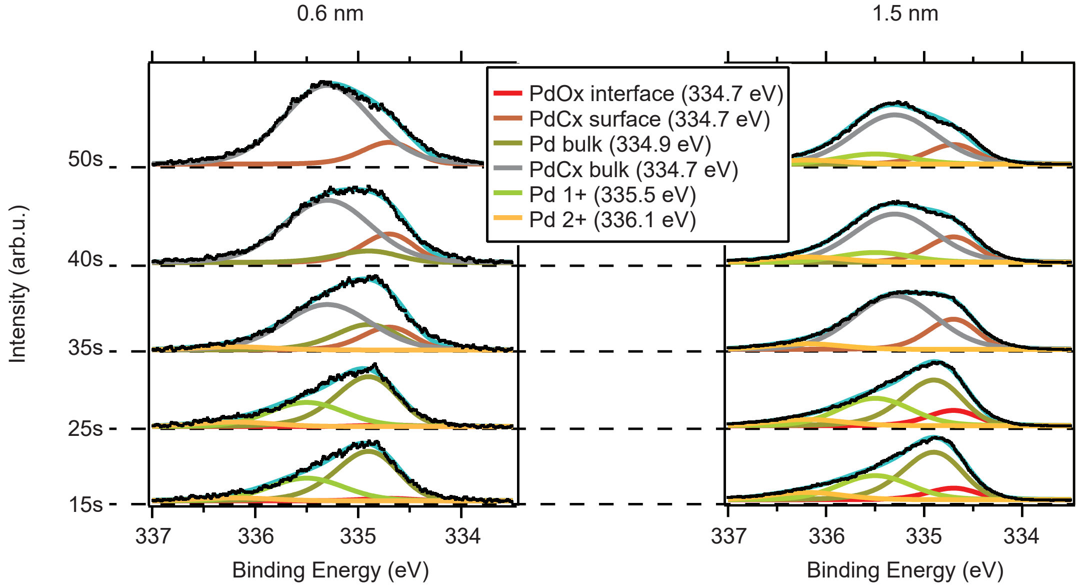

Figure S8: Measured Pd $3d_{\frac{5}{2}}$ spectra at 0.6 nm and 1.5 nm probing depth with possible curve fits and the peak positions of the used peaks with possible interpretations. Pd bulk and Pd surface were fitted with asymmetric Voigt peaks. For the PdCx surface component we found no tabulated values, thus, assigning this fitted component to PdCx is a guess based on the fact that we observe a bulk and surface carbide component in the C *1s* spectra and the fact that the component at $334.7\,\mathrm{eV}$ decreases in intensity when probing deeper layers. Now, the curve fitted spectra at $15\,\mathrm{s}$ and $25\,\mathrm{s}$ agree with the literature on thin oxides while the curve fits for later times shows increasingly thick PdCx layers.

Supplementary References

[1] J. L. Campbell and Tibor Papp. "Widths of the atomic K-N7 levels". In: *Atomic Data and Nuclear Data Tables* 77 (1 2001), pp. 1–56. ISSN: 0092640X. DOI: 10.1006/adnd.2000.0848.

[2] Jan Knudsen et al. "Catalysis in Frequency Space: Resolving Hidden Oscillating Minority Phases and Their Catalytic Properties". In: *ACS Catalysis* 15.3 (2025), pp. 1655–1662. DOI: 10.1021/acscatal.4c06355. eprint: https://doi.org/10.1021/acscatal.4c06355. URL: https://doi.org/10.1021/acscatal.4c06355.

[3] Ulrike Küst et al. "Comparing phase sensitive detection and Fourier analysis of modulation excitation spectroscopy data exemplified by Ambient Pressure X-ray Photoelectron Spectroscopy". In: *Surface Science* 751 (2025), p. 122612. ISSN: 0039-6028. DOI: https://doi.org/10.1016/j.susc.2024.122612. URL: https://www.sciencedirect.com/science/article/pii/S0039602824001638.

[4] Jan Knudsen et al. "Stroboscopic operando spectroscopy of the dynamics in heterogeneous catalysis by event-averaging". In: *Nature Communications* 12 (1 2021), p. 6117. ISSN: 2041-1723. DOI: 0.1038/s41467-021-26372-y. URL: https://doi.org/10.1038/s41467-021-26372-y.

[5] Xiansheng Li et al. "Role of Water on the Structure of Palladium for Complete Oxidation of Methane". In: *ACS Catalysis* 10 (10 May 2020), pp. 5783–5792. ISSN: 21555435. DOI: 10.1021/acscatal.0c01069.

[6] Jinwon Oh et al. "Palladium Catalysts for Methane Oxidation: Old Materials, New Challenges". In: *Accounts of Chemical Research* 57 (1 Jan. 2024), pp. 23–36. ISSN: 15204898. DOI: 10.1021/acs.accounts.3c00454.

[7] Shengnan Yue et al. "Redox dynamics and surface structures of an active palladium catalyst during methane oxidation". In: *Nature Communications* 15.1 (2024), p. 4678. ISSN: 2041-1723. DOI: https://doi.org/10.1038/s41467-024-49134-y.

[8] Ryo Toyoshima et al. "In Situ Ambient Pressure XPS Study of CO Oxidation Reaction on Pd(111) Surfaces". In: *The Journal of Physical Chemistry C* 116.35 (2012), pp. 18691–18697. DOI: 10.1021/jp301636u. eprint: https://doi.org/10.1021/jp301636u. URL: https://doi.org/10.1021/jp301636u.

[9] Detre Teschner et al. "Role of Hydrogen Species in Palladium-Catalyzed Alkyne Hydrogenation". In: *The Journal of Physical Chemistry C* 114.5 (2010), pp. 2293–2299. DOI: 10.1021/jp9103799. eprint: https://doi.org/10.1021/jp9103799. URL: https://doi.org/10.1021/jp9103799.

[10] D. Teschner et al. "High-pressure X-ray photoelectron spectroscopy of palladium model hydrogenation catalysts.: Part 1: Effect of gas ambient and temperature". In: *Journal of Catalysis* 230.1 (2005), pp. 186–194. ISSN: 0021-9517. DOI: https://doi.org/10.1016/j.jcat.2004.11.036. URL: https://www.sciencedirect.com/science/article/pii/S0021951704005767.

[11] Detre Teschner et al. "Alkyne hydrogenation over Pd catalysts: A new paradigm". In: *Journal of Catalysis* 242.1 (2006), pp. 26–37. ISSN: 0021-9517. DOI: https://doi.org/10.1016/j.jcat.2006.05.030. URL: https://www.sciencedirect.com/science/article/pii/S0021951706002016.
